# Physiotherapy Capabilities in the Health Care of Adult Patients at Increased Risk of Osteoporotic Fractures: A Scoping Review Protocol

**DOI:** 10.1002/msc.70125

**Published:** 2025-05-22

**Authors:** Fiona Blackman, Nicola Walsh, Zoe Paskins

**Affiliations:** ^1^ Integrated Musculoskeletal Service Solihull Community University Hospitals Birmingham NHS Foundation Trust Birmingham UK; ^2^ Centre for Health and Clinical Research University of the West of England Bristol UK; ^3^ School of Medicine Keele University Keele UK; ^4^ Haywood Academic Rheumatology Centre Midlands Partnership University NHS Foundation Trust Stoke‐on‐Trent UK

**Keywords:** attributes, bone health, competencies, knowledge, osteoporosis, physiotherapist, skills

## Abstract

**Objectives:**

This scoping review will explore and map the extent and type of literature related to physiotherapy capabilities in the health care of adult patients at increased risk of osteoporotic fractures and identify gaps in the literature.

**Background:**

With an ageing population, osteoporosis and fractures and their associated costs are increasing. Unfortunately, many people with, or at increased risk of, osteoporotic fractures remain undiagnosed and untreated. The scope of physiotherapy has expanded over recent years with enhanced, advanced and consultant roles. This presents opportunities for physiotherapists to help reduce the osteoporosis care gap. Clarifying capabilities is important to ensure successful implementation of new roles and development of holistically competent practitioners.

**Eligibility Criteria:**

Literature will be included if it relates to capabilities of qualified physiotherapists involved in the health care of adult patients (aged 18 and over) at increased risk of osteoporotic fractures in any clinical setting. All published and non‐published literature to include research studies, editorials, and grey literature will be considered.

**Methods:**

A scoping review will be carried out in accordance with JBI guidelines. Peer‐reviewed databases including MEDLINE, EMBASE, EMCARE, CINAHL, SCOPUS, Cochrane, PEDro and ProQuest will be searched for literature between 2003 and 2025, alongside a comprehensive search for grey literature (including reports and government publications) from additional sources to ensure a broad representation of available evidence. Source selection will use the PRISMA‐ScR flow chart against agreed eligibility criteria. Data extraction will be mapped out in tabular and/or diagrammatic format with a supporting narrative synthesis.

**Trial Registration:**

https://doi.org/10.17605/OSF.IO/47UYE

## 1 Introduction

Fractures that result from no or low‐energy trauma are a sign of underlying osteoporosis (World Health Organisation 2024a). In Western Europe, approximately one in three women and one in five men aged over 50 will experience an osteoporotic fracture in their lifetime (Borgström et al. 2020), with a significant long‐term impact on quality of life and mortality (Rizkallah et al. 2020). The cost of these fractures to the National Health Service (NHS) in the United Kingdom (UK) is approximately £4 billion annually and is predicted to rise along with an ageing population (Clynes et al. 2020).

In the last two decades, there have been advances in osteoporosis care, particularly in the measurement of bone mineral density, assessment of fracture risk, and development of interventions to reduce fracture risk (Curtis et al. 2022). Clinical guidelines highlight the importance of the early identification and management of patients with, or at increased risk of osteoporotic fractures (Gregson et al. 2022; LeBoff et al. 2022). However, despite having these clear guidelines and the availability of safe and effective pharmacotherapies that reduce the risk of fractures (Reid and Billington 2022), a well‐documented osteoporosis care gap persists (Ralston et al. 2022). Reasons for this are multifactorial and include at‐risk patients not being screened to establish fracture probability, not being educated about fracture prevention, not being offered treatment to reduce fracture risk following osteoporotic fractures, and not taking bone strengthening medication as recommended (LeBoff et al. 2022; Wang and Seibel 2024).

Physiotherapists have a well‐established role in delivering treatment interventions such as resistance and weight bearing exercises, balance training, and falls prevention advice for patients who have already been diagnosed with osteoporosis or osteoporotic fractures (Avin et al. 2022; Hartley et al. 2022). However, the scope of physiotherapy has been expanding over time and now includes enhanced, advanced and consultant level practice roles (NHS England 2023b). Physiotherapists working at these levels draw on a substantial knowledge base, understanding and experience to autonomously manage complexity and risk (Leary 2022). They act as a first point of contact for patients presenting with undifferentiated and undiagnosed conditions, and are trained to assess, diagnose, and develop management plans (Chance‐Larsen et al. 2019). Evidence shows that this care is of equal or better quality and lower cost than that provided by doctors in the assessment and management of musculoskeletal conditions (Walsh et al. 2024). The scope of practice varies but includes requesting diagnostic imaging, ordering blood tests, and independently prescribing (or de‐prescribing) medication to support patient care (Walsh et al. 2024). This offers opportunities for physiotherapists to help reduce the osteoporosis care gap, not just through exercise prescription and falls risk reduction but also through osteoporosis and fracture risk evaluation, identification of fractures, referral for appropriate investigations such as bone density scans, and prescription of drug treatments to improve bone health.

Physiotherapists have demonstrated the ability to effectively manage patients with musculoskeletal disorders in various secondary care settings, including orthopaedic clinics, rheumatology clinics, and emergency departments (Vedanayagam et al. 2021), as well as in primary care (Walsh et al. 2024). Patients with, or at risk of, osteoporotic fractures will regularly be encountered in these settings, often opportunistically when they present with other musculoskeletal conditions. However, there is very little reference made in the literature to the role of enhanced, advanced and consultant physiotherapists specifically in osteoporosis health care.

Clarifying professional competencies, capabilities and boundaries is important to ensure successful implementation of new roles and development of holistically competent practitioners (Scodras et al. 2022). Lack of clarity in level of practice can lead to difficulties for patients, other healthcare professionals, institutions, the media and the public in comprehending what different physiotherapy roles truly entail (Tawiah et al. 2021). This can result in physiotherapists being inappropriately used, thus not effectively utilising their expert skills sets and undermining their productivity and achievement of full scope of practice (Moffatt et al. 2018). Both competency and capability describe the knowledge, skills, and attributes that individuals bring to the workplace, but competency relates to practice in stable environments with familiar problems, whereas capability goes beyond this to include the ability to work flexibly and creatively in complex or unpredictable situations (Skills for Health 2020).

Competency and capability frameworks are common in social and healthcare contexts to identify knowledge and/or skill gaps and to guide workforce training and education (Taylor et al. 2025). Various frameworks have been published in the UK to ensure national consistency in level of practice across physiotherapy roles, such as First Contact Physiotherapy Practitioners (Health Education England 2021), Advanced Practice Physiotherapists (NHS England 2017), and Consultant Physiotherapists (NHS England 2023a), but these do not include condition‐specific capabilities.

In 2021, the Rheumatology Physiotherapy Capabilities Framework was launched to provide a formal description of specialist physiotherapy capabilities that ensure safe, effective assessment and management of people with underlying undiagnosed or diagnosed rheumatological conditions (British Society for Rheumatology 2021). The development of this document included a scoping process to identify rheumatology specific knowledge, skills and attributes encompassed by rheumatology specialist physiotherapists and physiotherapists in screening roles (Gregory et al. 2021). However, the document is heavily focussed on inflammatory conditions rather than bone health, with only four mentions of the word osteoporosis in the 38‐page document. More recently, Jackson et al. (2024) carried out a modified Delphi method to produce rigorously defined criteria for a clinician with competency in bone health. However, the study was not specific to physiotherapists, and the characteristics included factors more relevant to medically trained specialist bone health clinicians. It has also been suggested that the study may have been subject to participation bias due to the absence of a diverse panel with various backgrounds and expertise in bone health (Ganda et al. 2024).

A preliminary search of PROSPERO, MEDLINE, the Cochrane Database of Systematic Reviews, JBI Evidence Synthesis, Google Scholar and the Open Science Framework conducted in March 2025 identified no existing or ongoing scoping or systematic reviews identifying physiotherapy competencies or capabilities in osteoporosis. Therefore, the aims of this scoping review are to explore and map the extent and type of literature related to physiotherapy capabilities in the health care of adult patients at increased risk of osteoporotic fractures and identify gaps in the literature. The findings could help guide further research to establish an osteoporosis‐specific capability framework for physiotherapists.

## 2 Review Question

What physiotherapy capabilities are identified in the literature in relation to the provision of health care for adult patients at increased risk of osteoporotic fractures in all clinical settings?

## 3 Objectives

The review objectives are:

1. To explore and map the extent and type of literature related to physiotherapy capabilities in the provision of health care for adult patients at increased risk of osteoporotic fractures in all clinical settings.
2. To identify any gaps in the literature related to physiotherapy capabilities in the provision of health care for adult patients at increased risk of osteoporotic fractures in all clinical settings.

## 4 Methods

A scoping review has been chosen because it is best for exploring the breadth or extent of the literature, mapping and summarising the evidence, and informing future research (Tricco et al. 2016). Additionally, scoping reviews are inclusive and allow peer‐reviewed, non‐peer‐reviewed, and grey literature (Arksey and O'Malley 2007; Munn et al. 2018). The proposed scoping review has been planned in accordance with the JBI methodology for scoping reviews (Peters et al. 2020). The final manuscript will follow the Preferred Reporting Items for Systematic Review and Meta‐Analyses Extension for Scoping Reviews (PRISMA‐ScR) reporting guideline (Tricco et al. 2018).

### 4.1 Eligibility Criteria

The population, concept, context (PCC) framework has been used to develop the review question and objectives and inform the eligibility criteria (Peters et al. 2020).

### 4.2 Population

This scoping review will consider the literature relating to qualified physiotherapists at all levels of practice.

### 4.3 Concept

The review will include literature that describes physiotherapy capabilities (including beliefs and/or perceptions about capabilities) related to all aspects of osteoporosis health care. The broad term ‘capability’ will be used to encompass knowledge, skills, and attributes, including the ability to be competent in situations which may be complex or unpredictable (Skills for Health 2020).

### 4.4 Context

The review will consider literature related to any clinical setting where physiotherapists are involved in the health care of adult patients (aged 18 and over) at increased risk of osteoporotic fractures. This may include acute hospitals, primary care centres, private practices, and community settings.

### 4.5 Types of Sources

A wide range of sources will be examined for this review, including peer‐reviewed academic journal articles (research studies and reviews of all designs and case reports) and editorials/viewpoints. Grey literature including dissertations, theses, conference papers, research reports, articles that have not yet been peer‐reviewed (pre‐prints), and official reports and publications from physiotherapy associations, health regulatory bodies and government agencies will also be considered. Literature from 2003 to 2025 will be included to reflect current physiotherapy scope of practice, following the introduction of Consultant Physiotherapists in the UK in 2003, demonstrating the highest level of physiotherapy clinical practice (Chartered Society of Physiotherapy 2016). Literature from high‐ and upper‐middle‐income countries/territories will be included to reflect regions that are more likely to have similar healthcare systems (World Health Organisation 2024b), as well as direct access to physiotherapy (World Physiotherapy 2024). Literature that is not written in the English language will be excluded due to financial constraints for translation services. Literature will only be considered if the full text is available. The inclusion and exclusion criteria are summarised in Table 1.

**TABLE 1:** Summary of scoping review inclusion and exclusion criteria.

	Inclusion criteria	Exclusion criteria
Population	Qualified physiotherapists	Multi‐professional groups including physiotherapists without stratified data/information per discipline Undergraduate physiotherapy students
Concept	Physiotherapy capabilities (including beliefs and/or perceptions about capabilities) related to all aspects of osteoporosis health care, including knowledge, skills and attributes	Physiotherapy capabilities that are not specific to osteoporosis health care Physiotherapy capabilities that relate to undergraduate education
Context	Any clinical setting where physiotherapists are involved in the health care of adult patients aged 18 and over at increased risk of osteoporotic fractures	Non‐clinical settings
Types of sources	Peer reviewed academic journal articles (research studies and reviews of all designs and case reports) and editorials/viewpoints Dissertations, theses, conference papers, research reports and articles that have not yet been peer‐reviewed (pre‐prints) Official reports and publications from physiotherapy associations, health regulatory bodies and government agencies	Abstracts for which full text is not available Protocol studies Conference proceedings without sufficient data for extraction
Other	Literature from 2003 to 2025 Literature from high and upper‐middle‐income countries/territories Literature written in the English language	Literature prior to 2003 Literature from lower‐middle and low‐income countries/territories Literature written in non‐English language

### 4.6 Search Strategy

The search strategy has been developed in consultation with an academic librarian, as recommended by Peters et al. (2022). The search will aim to locate both published and unpublished literature, following a three‐stage approach (Pollock et al. 2021).

#### 4.6.1 Stage 1

As part of this protocol, an initial limited search of MEDLINE (Ovid) and CINAHL was undertaken to identify articles on the topic. Keywords and phrases in the titles and abstracts of relevant articles and the associated index terms were used to inform an initial search strategy. This was pilot tested on CINAHL (Supporting Information S1: Appendix I).

#### 4.6.2 Stage 2

The full search strategy will be carried out on a broad range of relevant databases including MEDLINE (Ovid), EMBASE (Ovid), EMCARE (Ovid), CINAHL (EBSCO), SCOPUS, Cochrane, PEDro, and ProQuest (including Dissertations and Theses). The search strategy, including all identified keywords and index terms, will be adapted for each included database as required.

#### 4.6.3 Stage 3

The reference list of all included sources of evidence will be systematically screened for additional relevant literature. Sources of grey literature to be searched will include the King's Fund (https://koha.kingsfund.org.uk/) and Trip (https://www.tripdatabase.com/) websites. A systematic search will also be conducted via the websites of national physiotherapy associations, health regulatory bodies and government agencies in the UK, Ireland, Canada, the United States of America, Australia and New Zealand, to reflect countries that have direct access to physiotherapy where English is the first language (World Physiotherapy 2024). The search strategy for each website with all key words used will be fully documented and reported the following completion of the review.

### 4.7 Source of Evidence Selection

During the search, all identified records will be collated and uploaded into Mendeley Reference Manager (www.mendeley.com) for storage and organisation. Duplicates will be deleted, and references will then be exported to the Rayyan application (www.rayyan.ai) to facilitate independent record selection by the reviewers.

A pilot test of the eligibility criteria will be performed by two independent reviewers on a random sample of 25 titles and abstracts to ensure an interrater agreement of at least 75%, as suggested by the JBI framework (Aromataris et al. 2024). If required, reviewers will have additional discussion and modify the eligibility criteria until this interrater agreement threshold is achieved.

The two reviewers will then independently evaluate the remaining titles and abstracts against the inclusion criteria. Following this, potentially relevant sources will be retrieved in full and assessed in detail for further review against the inclusion criteria by the same two independent reviewers. Authors will be contacted for further information, if required. If no response has been received within 4 weeks, the authors will be categorised as unreachable and their publication will be excluded if the information provided by the article is not sufficient. All reasons for exclusion of any ineligible sources of evidence at the full text stage will be recorded and reported. Any disagreements will be resolved by consensus or by the decision of a third reviewer.

**FIGURE 1:**
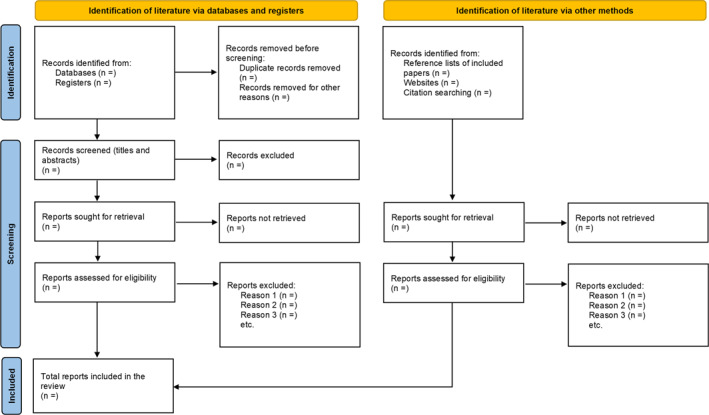
PRISMA flowchart of the scoping review.

The search results and source inclusion process will be reported in full in the final scoping review and presented in a Preferred Reporting Items for Systematic Reviews and Meta‐analyses extension for scoping review (PRISMA‐ScR) flow diagram (Tricco et al. 2018) (Figure 1).

### 4.8 Data Extraction

Data will be extracted from the literature included in the scoping review into Microsoft Excel using a modified version of the JBI template source of evidence details, characteristics and results extraction instrument (Aromataris et al. 2024). A draft data extraction form is provided (Supporting Information S2: Appendix II). Extracted data regarding capabilities will be mapped to six domains (screening, investigations, physiotherapy interventions, condition management, medication management, and non‐clinical capabilities) and allocated as a knowledge (K), skill (S), attribute (A), or any combination of these three, in line with the Rheumatology Physiotherapy Capabilities Framework (British Society for Rheumatology 2021). Authors will be contacted to request missing or additional data if required. One reviewer will extract the data initially. This will be checked and added to as felt necessary by a second reviewer. Any disagreements that arise will be resolved through discussion or with the assistance of a third reviewer. The draft data extraction form will be piloted by the first and second reviewers on a single article on each evidence source prior to commencing data charting and any modifications will be documented. As a scoping review is an iterative process, the data extraction form will be modified and revised as necessary during the process of the review in response to the emergent findings. Any modifications will be detailed in the final scoping review.

### 4.9 Data Analysis and Presentation

The extracted data will be grouped and presented in tabular and/or diagrammatic format in a manner that aligns with the review question and objectives of this scoping review. A table will be developed to present the capabilities, including the knowledge, skills, and attributes that are identified in the literature that support physiotherapists in providing health care for adult patients at increased risk of osteoporotic fractures. A narrative summary will accompany the tabulated and/or charted results and any gaps in the literature will be highlighted.

## 5 Conclusion

This will be the first scoping review to explore the current literature related to physiotherapy capabilities in the health care of adult patients at increased risk of osteoporotic fractures. Any gaps in the literature will be discussed. The findings could help guide further research to establish an osteoporosis core capability framework for physiotherapists working at all levels of practice to ensure their full potential can be realised in helping to reduce the osteoporosis care gap.

## Author Contributions

**Fiona Blackman:** conceptualization, methodology, validation, writing – original draft, writing – review and editing, visualization, project administration. **Nicola Walsh:** conceptualization, methodology, validation, writing – review and editing, visualization, project administration, project supervision. **Zoe Paskins:** conceptualization, methodology, validation, writing – review and editing, visualization, project administration, project supervision. All authors approved the final version of the manuscript.

## Ethics Statement

The authors have nothing to report.

## Conflicts of Interest

The authors declare no conflicts of interest.

## Supporting information

Table S1

Table S2

## Data Availability

Data sharing is not applicable to this article as no new data were created or analyzed in this study.
