# Supplementary material for: Physiotherapy Capabilities in the Health Care of Adult Patients at Increased Risk of Osteoporotic Fractures: A Scoping Review Protocol
Source: Musculoskeletal Care. 2025 May 22;23(2):e70125. doi: 10.1002/msc.70125 (PMC12097966; doi:10.1002/msc.70125)
Supplement: Supplementary file 1 — Table S1 [file MSC-23-e70125-s002.docx]

**Appendix I: Search strategy**

CINAHL (EBSCO)

Search conducted on 26/03/2025

| Search | Query | Records retrieved |
| --- | --- | --- |
| #1 | physiotherap* OR “physical therap*” OR PT OR “first contact physiotherapy practitioner*” OR FCPP OR “first contact practitioner*” OR FCP OR “extended scope practitioner*” OR ESP OR “allied health professional*” OR AHP  *Limit to title or abstract | 39,092 |
| #2 | Capabilit* OR competenc* OR knowledge OR skill* OR attribute* OR behaviour* OR attitude* OR role OR activit* OR task* OR scope  *Limit to title or abstract | 1,184,936 |
| #3 | Osteoporo* OR osteopeni* OR “bone loss” OR “bone density” OR “bone mineral density” OR BMD OR “bone fragility” OR “bone health” OR “fragility fracture*” OR “fracture risk*”  *Limit to title or abstract | 32,868 |
| #4 | #1 AND #2 AND #3 | 137 |
| Limited to English language, abstract available, publication date 2003-2025 | | |
