# Supplementary material for: Physiotherapy Capabilities in the Health Care of Adult Patients at Increased Risk of Osteoporotic Fractures: A Scoping Review Protocol
Source: Musculoskeletal Care. 2025 May 22;23(2):e70125. doi: 10.1002/msc.70125 (PMC12097966; doi:10.1002/msc.70125)
Supplement: Supplementary file 2 — Table S2 [file MSC-23-e70125-s001.docx]

**Appendix II: Data extraction instrument**

| **Title:**  Physiotherapy capabilities in the health care of adult patients at increased risk of osteoporotic fractures: a scoping review protocol | | | |
| --- | --- | --- | --- |
| **Does the study meet the eligibility criteria?** | **Yes** | **No** | |
| **Evidence Source Details and Characteristics** | | | |
| Title |  | | |
| Author(s) |  | | |
| Year |  | | |
| Country of origin |  | | |
| Source details (name of journal, website, etc.) |  | | |
| Source type |  | | |
| Aims/purpose |  | | |
| **Population** | | | |
| Physiotherapist level of practice |  | | |
| **Context** | | | |
| Care setting (primary care, secondary care, private practice, community, etc.) |  | | |
| Patient group (age, gender, fracture risk factors, history of fracture, etc.) |  | | |
| **Concept** | | | |
|  | Capabilities Identified | | Knowledge, Skills, Attributes (KSA) |
| **Screening**  (identification of those at risk of fracture and osteoporosis; identification of low/no trauma fractures – particularly spinal fractures, etc.) |  | |  |
| **Investigations** (fracture risk calculation, bone density scans, falls assessment, etc.) |  | |  |
| **Physiotherapy interventions**  (exercise therapy, reducing falls risk, management of fracture symptoms, etc.) |  | |  |
| **General condition management**  (identification and treatment of secondary causes, lifestyle changes, patient education, etc.) |  | |  |
| **Medication management**  (drug treatments for fracture risk reduction) |  | |  |
| **Non-clinical**  (research, education, and leadership) |  | |  |
| **Other** |  | |  |
| **Key Findings** | | | |
| Summary |  | | |
